# Single-cell sequencing dissects the transcriptional identity of activated fibroblasts and identifies novel persistent distal tubular injury patterns in kidney fibrosis

**DOI:** 10.21203/rs.3.rs-2880248/v1

**Published:** 2023-05-17

**Authors:** Valeria Rudman-Melnick, Mike Adam, Kaitlynn Stowers, Andrew Potter, Qing Ma, Saagar M. Chokshi, Davy Vanhoutte, Iñigo Valiente-Alandi, Diana M. Lindquist, Michelle L. Nieman, J. Matthew Kofron, S. Steven Potter, Prasad Devarajan

**Affiliations:** Cincinnati Children’s Hospital Medical Center; Cincinnati Children’s Hospital Medical Center; Cincinnati Children’s Hospital Medical Center; Cincinnati Children’s Hospital Medical Center; Cincinnati Children’s Hospital Medical Center; Cincinnati Children’s Hospital Medical Center; Cincinnati Children’s Hospital Medical Center; Cytokinetics (United States); Cincinnati Children’s Hospital Medical Center; University of Cincinnati; Cincinnati Children’s Hospital Medical Center; Cincinnati Children’s Hospital Medical Center; Cincinnati Children’s Hospital Medical Center

## Abstract

Examining kidney fibrosis is crucial for mechanistic understanding and developing targeted strategies against chronic kidney disease (CKD). Persistent fibroblast activation and tubular epithelial cell (TEC) injury are key CKD contributors. However, cellular and transcriptional landscapes of CKD and specific activated kidney fibroblast clusters remain elusive. Here, we analyzed single cell transcriptomic profiles of two clinically relevant kidney fibrosis models which induced robust kidney parenchymal remodeling. We dissected the molecular and cellular landscapes of kidney stroma and newly identified three distinctive fibroblast clusters with “secretory”, “contractile” and “vascular” transcriptional enrichments. Also, both injuries generated failed repair TECs (frTECs) characterized by decline of mature epithelial markers and elevation of stromal and injury markers. Notably, frTECs shared transcriptional identity with distal nephron segments of the embryonic kidney. Moreover, we identified that both models exhibited robust and previously unrecognized distal spatial pattern of TEC injury, outlined by persistent elevation of renal TEC injury markers including Krt8, while the surviving proximal tubules (PTs) showed restored transcriptional signature. Furthermore, we found that long-term kidney injuries activated a prominent nephrogenic signature, including *Sox4* and *Hox* gene elevation, which prevailed in the distal tubular segments. Our findings might advance understanding of and targeted intervention in fibrotic kidney disease.

## Introduction

Fibrosis is a key underlying process in CKD, resulting in a progressive functional decline with high prevalence, morbidity and mortality.^1–3^ While early fibrotic response is essential for injury recovery,^4^ excessive extracellular matrix (ECM) production leads to renal parenchymal fibrotic remodeling.^5^ Since existing therapeutic options remain merely supportive^6^ and advanced CKD might result in end-stage kidney disease (ESKD) requiring lifelong dialysis or transplant,^7^ mechanistic understanding of kidney fibrosis is paramount.

Aberrant injury induced fibroblast activation and appearance of myofibroblasts is a crucial pathologic contributor to kidney fibrosis.^8–11^ Yet, existing approaches to ECM-producing renal cell population targeting remain controversial, due to the nonspecific expression of currently used markers such as *Acta2*, *Col1a1* and *Vim*.^12–22^ A recent study examined human CKD and murine UUO induced renal fibrosis; however, defining kidney fibroblasts via ECM genes or *Pdgfrβ* might not allow for specific capturing of all heterogeneous stromal populations.^12,23,24^

Unresolved TEC injury and pro-fibrotic changes represent another key CKD landmark.^25^ Multiple studies^26–30^ have examined healthy and abnormal PT transcriptional and translational profiles due to their crucial role in kidney metabolism and injury. Recent scRNA-seq analysis identified and pharmacologically tested molecular pathways involved in PT repair.^31^ Also, several studies revealed new “repairing”, “injured” and “failed repair” PT states appearing during dynamic kidney injury response.^32–34^ “Failed repair” PTs displayed dedifferentiated proinflammatory and profibrotic transcriptional states associated with CKD progression. However, little is known about the transcriptional signatures of distal nephron tubular segments, despite our recent report showing robust renal developmental program reactivation in distal segments.^29^

Here, we dissected the molecular and cellular events defining two clinically relevant murine models of kidney fibrosis. By combining multiple scRNA-seq replicate analysis with thorough validation, we identified three fibroblast clusters with distinctive transcriptional signatures along with persistent distal spatial pattern of long-term kidney tubular epithelial remodeling, injury and renal developmental program reactivation.

## Results

### Kidney fibrosis models exhibit proximal tubular loss, functional decline and novel cellular clusters.

**Figure 1:**
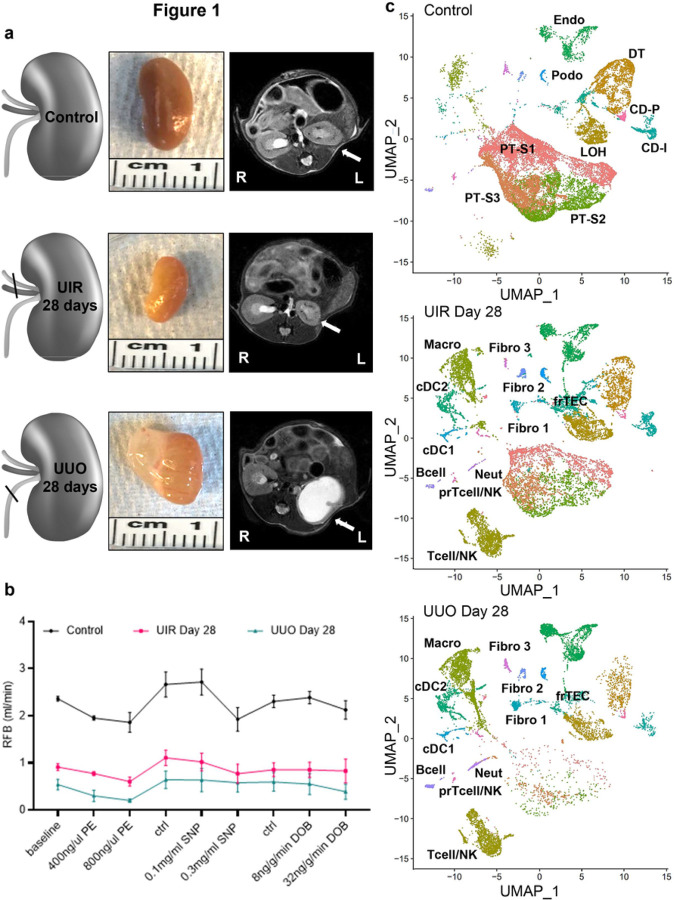
Ischemia/reperfusion and obstruction induced models of CKD elicit dramatic proximal tubular loss, kidney functional decline and novel cellular clusters. (a) Schemes of injury models (left), macroscopic (middle) and MRI (right) images of normal control, UIR and UUO day 28 kidneys. Kidneys are pointed to with white arrows. R, right, L, left. (b) Renal blood flow (RBF) at baseline, with vasoconstrictive (PE, phenylephrine), vasodilative (SNP, sodium nitroprusside) and inotropic agent (DOB, dobutamine). Agents administered at 0.1ul/min/gBW, Ctrl – control interval between treatments, n=3–4 per group, mean ± SD. **P≤ 0.01, ***P ≤ 0.001, ****P ≤ 0.0001 compared to control, Student’s t test. (c) UMAPs show renal cell populations in the control, UIR and UUO kidneys (n=3–5 per group). Clusters are distinguished by different colors. PT, proximal tubules, S1/2/3 – segment 1/2/3, LOH, loop of Henle, DT, distal tubule, CD-P, collecting duct principal, CD-I, collecting duct intercalated, Podo, podocytes, Endo, endothelial, Macro, macrophages, Neutro, neutrophils, cDC, conventional dendritic cells, NK, natural killer, prTcell, proliferating Tcell, Fibro, fibroblasts, frTEC, failed repair tubular epithelial cells.

**Figure 2:**
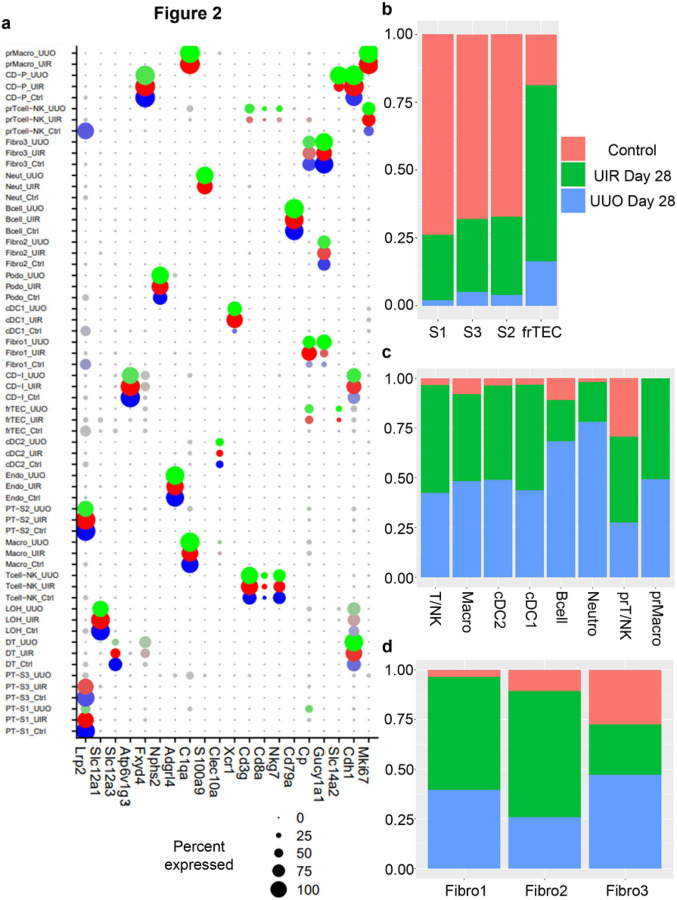
scRNA-seq dissects molecular and cell type proportion changes in the UIR and UUO models of renal fibrosis. (a) Dot plot of cell type-specific expression of marker genes for manually annotated clusters. Dot size denotes percentage of cells expressing the marker. Color intensity represents average gene expression values. Complete marker gene list is presented in Supplementary table S1. (b) Relative cell subset proportion in the control (salmon), UIR (green) and UUO (blue) kidneys. Cell subset proportion change is shown relative to the listed conditions.

We established two clinically relevant kidney fibrosis models induced by UIR and UUO.^35–37^ At day 28 post-injury, both models exhibited key CKD features including renal parenchymal loss and blood flow decline^38^ (Fig. 1a and b, Supplementary Figure S1). Then, 10x Chromium scRNA-seq was carried out on the control, UIR and UUO kidney suspensions. Kidney cell populations were identified using unsupervised Uniform Manifold Approximation and Projection (UMAP) dimension reduction,^39^ and datasets were cleansed of potential doublets, ambient RNA and cells with high mitochondrial and hemoglobin components (Supplementary Figure S2–4). Controls exhibited podocyte (*Nphs1/2*), endothelial (*Adgrl4*) and epithelial clusters, including large PT S1 (*Slc5a2/12*), S2 (*Cyp2e1/4b1, Slc22a6*) and S3 (*Acy3, Slc27a2*) subpopulations and distal nephron tubular segments, represented by loop of Henle (*Slc12a1*), distal tubule (*Slc12a3*), collecting duct principal (*Fxyd4*) and intercalated (*Atp6v1g3*) cells (Fig. 1c, 2a, Supplementary Figure S5, Supplementary Table S1).^40^ UMAPs also revealed small myeloid clusters, including conventional dendritic (cDCs) 1 (*Xcir1, Clec9a*) and 2 (*Clec10a*),^41^ macrophages (*C1qa*) and neutrophils (*S100a9*), along with lymphoid B (*Cd79a*) and T/NK (*Cd3g, Cd8a, Nkg7*) cells.^42^

Both models caused remarkable cellular landscape changes, including dramatic PT decline (Fig. 1c, 2b, Supplementary figures S6 and S7). We previously showed that AKI induced PT loss and dedifferentiation are restored to the mature gene expression as AKI resolves.^29^ Instead, substantial S1-S3 PT decline persisted in our long-term models, indicating AKI-to-CKD progression. However, the surviving UIR and UUO Day 28 PTs returned to normal gene expression, except the UUO S3 segment which exhibited persistent low solute-linked carrier encoding gene levels and pro-inflammatory signature^33^ (*Lyz2, C1qa/b, Ifi30, S100a8/9*) (Supplementary figures S8 and S9). Moreover, scRNA-seq revealed a separate epithelial cluster, located between loop of Henle and distal tubule on the UMAP, which was present in the control and markedly expanded in the fibrotic kidneys (Fig. 1c, 2b). We labeled them as “failed repair TECs” (frTECs) due to the simultaneous expression of epithelial (*Slc14a2, Cdh1, Calb1*), stromal (*Cp, Vcam1*) and injury related (*Spp1, Krt8, Cryab*) genes (Fig. 2a, Supplementary figures S6 and S7, Supplementary table S1).

Both models also exhibited evident inflammatory expansion, represented by increased myeloid and lymphoid infiltration along with proliferating macrophages (Fig. 2c, Supplementary figures S10). While macrophages were the predominant immune fraction in the control, we observed Tcell/NK increase in the fibrotic kidneys. Moreover, scRNA-seq identified three separate fibroblast clusters, named “Fibro 1, 2 and 3” (Fig. 1c, 2d). We noted that Fibro 1 and 2 represented major stromal fractions of the fibrotic kidneys, while Fibro 3 cells were predominant in the controls (Supplementary figure S10). Validations on an independent control and injured mice cohorts verified substantial fibrotic remodeling, inflammatory expansion and PT loss at the RNA and protein levels (Supplementary figures S11 and S12). Thus, we successfully established two models of Kidney fibrosis exhibiting key CKD landmarks.^43^

### Prolonged kidney remodeling elicits pronounced epithelial-to-stromal crosstalk.

Since our previous report showed enhanced cell-to-cell interactions in AKI,^29^ we dissected the nature of intercellular crosstalk in advanced kidney injuries^44,45^ (Supplementary table S2). Normal kidneys exhibited epithelial-to-epithelial, epithelial-to-stromal and epithelial-to-immune interactions via calmodulin, cadherin, G-protein coupled receptor, beta-2-microglobulin and urokinase pathways (Supplementary figures S13–15). Both injury models caused enhanced communications (38538 ligand-receptor pairs in UIR and 44809 in UUO vs 24984 pairs in control), including epithelial-to-stromal crosstalk (Supplementary figures S16–19). scRNA-seq predicted that both injuries caused most notable increases in interactions between loop of Henle, collecting duct intercalated and PT S1 and 3 with Fibro 1 clusters, while the smallest number of receptor-ligand encoding gene pairs was found between collecting duct principal and stromal populations (Supplementary figure S20, Supplementary table S3). TECs in the fibrotic kidneys interacted with stromal cells via collagen (*Col4a1-Cd47/Itgav/Itgb1*), osteopontin (*Spp1-Cd44/Itgav/Itgb1*), metalloproteinase 2 (*Timp2-Itgb1*), vascular cell adhesion molecule 1 (*Vcam1-Itgb1/b7/a4*) pathways. We also observed that *Col18a1*, which encodes the alpha chain of type XVIII collagen implicated in ureteric tree development^46^ and which we previously reported in AKI,^29^ is elevated in the fibrotic kidney TECs, while it’s receptor encoding genes (*Gpc1/4, Itga5/b1*) are expressed in Fibro 1, 2 and 3 clusters, highlighting a putative interaction pathway present in both AKI and CKD models. scRNA-seq also showed that both models caused enhanced stromal-to-stromal and stromal-to-epithelial crosstalk (Supplementary figures S21 and 22). Fibroblasts interacted with each other and TECs via fibronectin, fibroblast growth factor 9/12, collagen, cadherin 1, transforming growth factor β (Tgfβ) and Wnt4 pathways. Of note, we revealed pronounced tubular-immune and stromal-immune communications, involving B cells, which is consistent with the recent report.^42^

### Advanced kidney remodeling exhibits three separate secretory, contractile and migratory fibroblast clusters with distinctive pathway enrichments.

**Figure 3:**
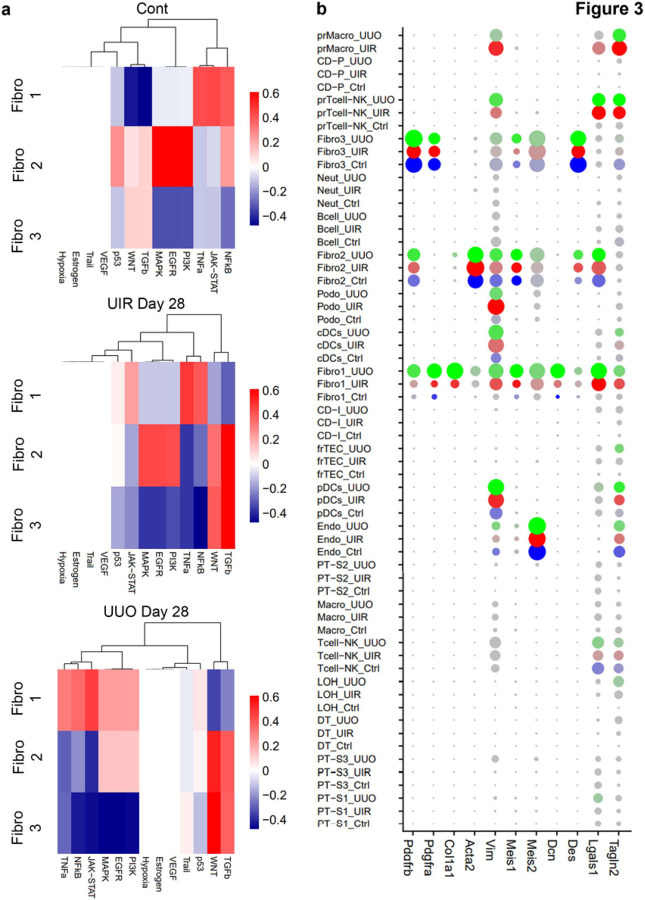
UIR and UUO elicit three transcriptionally distinctive fibroblast clusters. (a) Pathway RespOnsive GENes (PROGENy) for activity inference analysis of three fibroblast clusters in the control, UIR and UUO kidneys. Complete PROGENy analysis of all populations is present in the Supplementary figure S24. Expression levels are represented with color gradient. (b) Dot plot of cell type-specific expression of known fibrosis marker genes for manually annotated clusters. Dot size denotes percentage of cells expressing the marker. Color intensity represents average gene expression values.

Next, we aimed to dissect the molecular and cellular changes in the kidney stroma, induced by advanced injuries. Picrosirius red staining validated that both models repetitively induce remarkable stromal expansion and ECM deposition in the cortex and medulla (Supplementary figure S23). Using two independent scRNA-seq platforms, we repeatedly identified three distinctive fibroblast clusters in control and both fibrosis models (Fig. 1c, Supplementary figure S24), thus we further examined their unique transcriptional identities. Pathway responsive gene analysis (PROGENy)^47^ identified distinctions between Fibro 1, 2 and 3 clusters (Fig. 3a, Supplementary figure S25). Fibro 1 displayed elevation of TNFα, NFκB and JAK-STAT related genes while downregulating WNT and TGFβ. Fibro 2 was enriched for MAPK, EGFR and PI3K on the baseline and acquired WNT and TGFβ elevation in the injured kidneys. Fibro 3 exhibited WNT and TGFβ pathways enrichment on the baseline, which increased in both fibrosis models. Of note, cell division and death related p53 pathway was slightly upregulated in UIR and UUO Fibro 1 cluster and downregulated in Fibro 3 in both injured and control kidneys.

**Figure 4:**
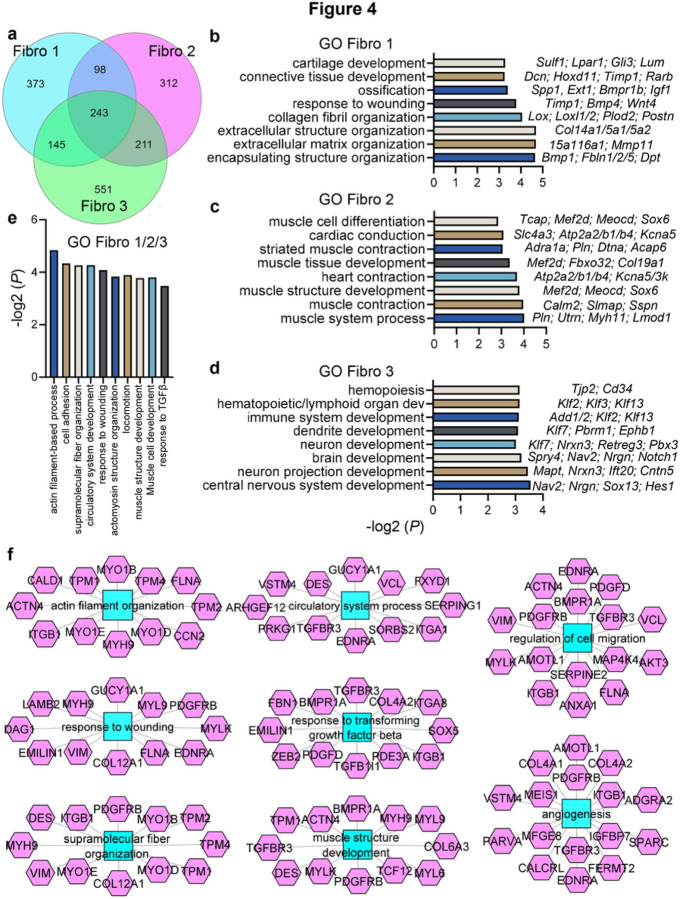
Three distinctive secretory, contractile and migratory fibroblast clusters emerge in advanced UIR and UUO. (a) Venn diagram displaying unique and shared Fibroblast 1, 2 and 3 marker genes. Complete lists of genes are presented in the Supplementary Table 4. (b-d) GO Biological process of fibroblast clusters unique marker genes vs other populations in control, UIR and UUO, – log2 (*P*). Representative genes are shown on the left for each biological process. Complete GO analysis is presented in the Supplementary table 4. (e) GO Biological process of fibroblast clusters 243 shared marker genes, – log2 (*P*). Complete GO analysis is presented in the Supplementary table 4. (f) ToppCluster analysis of biological processes enriched among 243 shared Fibro 1, 2 and 3 marker genes. Biological processes are highlighted with blue polygons, individual gene names – pink polygons.

Comparative analysis showed that genes unique for Fibro 1, including *Col1a1* and *Dcn*, were related to ECM production, cartilage development and ossification, which led us to labelling them as “secretory fibroblasts” (Fig. 3b and 4a-d, Supplementary table S4). Fibro 2 specific transcriptional identity included muscle development, differentiation and contraction related genes, including historical myofibroblast marker *Acta2*; thus, we identified them as “contractile”. Since Fibro 3 signature was enriched for hemopoiesis and immune system related biological processes along with neuron projection and dendrite development, we called them “migratory”. Fibro 3 was also the predominant fibroblast type in the normal kidney and exhibited the smallest fold increase in UIR and UUO, while Fibro 1 cluster was the most abundant in both injuries, exhibiting remarkable increase in the fibrotic kidneys (Fig. 2d, Supplementary figure S10c). In addition to the distinctions, three clusters had shared genes, related to TGFβ response, circulatory process and angiogenesis, cell migration and wound response^48^ (Fig. 4e and f). However, many markers, previously used to identify fibroblasts, including *Vim, Lgals1, Tagln2* and *Meis1/2*, exhibited non-specific expression among off-target kidney populations (Fig. 3b).

### Long-term kidney injury induced frTECs share transcriptional identity with embryonic and adult distal nephron tubular segments.

**Figure 5:**
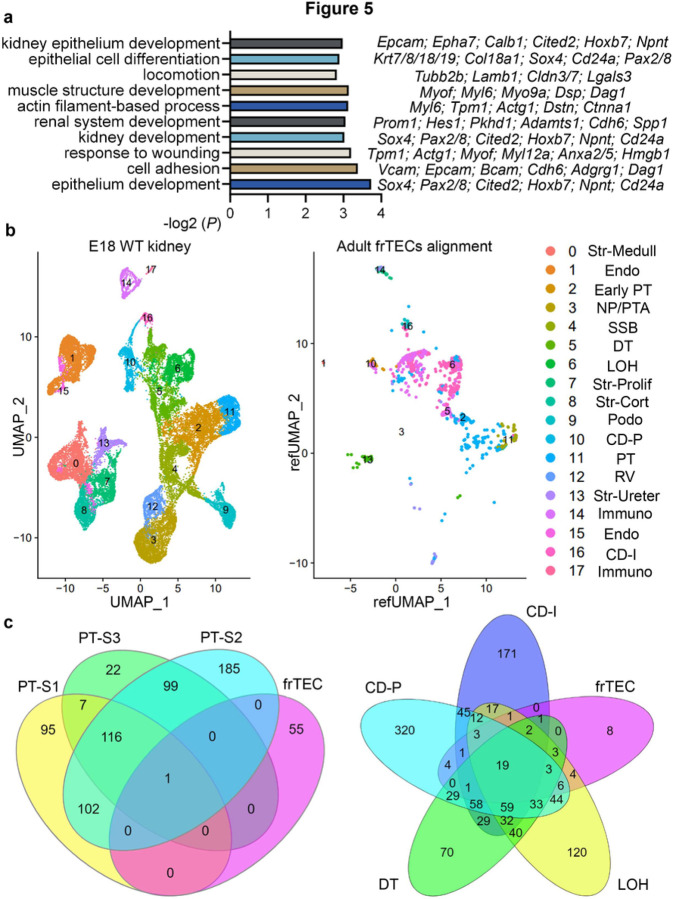
FrTECs show transcriptional similarity with embryonic and adult distal segments of the nephron tubule. (a) GO Biological process of “frTECs” marker genes vs other populations in control, UIR and UUO, – log2 (*P*). Names of the particular genes representing the biological process are listed on the left. Full biological process analysis and gene list is presented in the Supplementary table S5. (b) UMAPs show renal cell populations in the E18 WT kidney (left), adult frTECs alignment to the E18 WT kidney populations (middle) and E18 WT kidney cluster designation (right). WT, wild type. Data source for E18 WT kidney scRNA-seq: GSE214024. Complete marker gene list for E18 WT kidney is presented in the Supplementary table S6. (c) Venn diagrams displaying unique and shared PT S1–3, frTECs, loop of Henle, distal tubule and collecting duct principal and intercalated marker genes. Complete lists of genes are presented in the Supplementary Table 7.

Next, we questioned the transcriptional identity of frTECs, which exhibited minimal presence in the normal kidney and expanded presence in the fibrotic kidneys (Fig. 1c, 2b, Supplementary figure S7). Gene ontology (GO)^49^ analysis identified that frTECs were enriched for kidney and epithelium development (*Pax2/8, Cited2, Sox4, Cd24a, Cdh6, Npnt*)^50–52^ (Fig. 5a, Supplementary table S5), which indicates that these cells might be reverting to the dedifferentiated state as a result of injury.^53^ Moreover, frTECs exhibited signs of mesenchymal transcriptional signature,^25^ elevating genes related to locomotion, cell adhesion, muscle structure development and wounding. Thus, we asked whether this cluster originates from the distal or proximal segments of nephron tubule. scRNA-seq demonstrated that frTECs are located between the distal segments of nephron tubule, including loop of Henle, distal tubule and collecting duct, and the fibroblast clusters on the UMAP (Fig. 1c). Bioinformatic comparison of our datasets to the previously generated E18 WT kidney scRNA-seq data (GSE214024) revealed that adult frTECs mainly align with embryonic loop of Henle, distal tubule and collecting duct (Fig. 5b, Supplementary table S6). Moreover, comparison between adult tubular clusters produced substantial marker gene overlap between frTECs and distal nephron tubular segments, with only one gene shared with S1-S3 PTs (Fig. 5c, Supplementary table S7).

### Prolonged UIR and UUO exhibit persistent distal spatial patterns of tubular injury, while the remaining proximal tubules restore normal gene expression.

**Figure 6:**
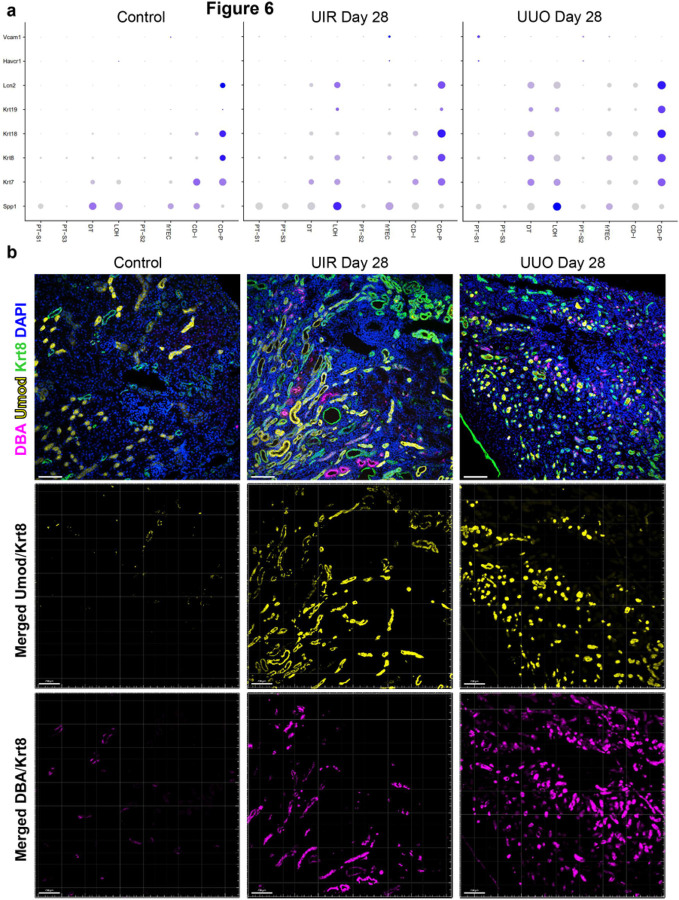
Long-term kidney parenchymal remodeling exhibits distal spatial pattern of tubular injury. (a) Dot plot of cell type-specific expression of renal epithelial injury markers for manually annotated clusters in the control, UIR and UUO kidney. Dot size denotes percentage of cells expressing the marker. Color intensity represents average gene expression values. (b) Upper panels - representative images of combined Krt8 IF (green), Umod (yellow), DAB (magenta) and DAPI (blue) in control, UIR and UUO kidneys. Original magnification, ×20, 0.86 μm/px zoom. Lower panels – merged Umod/Krt8 (middle panels, yellow) and DAB/Krt8 (lower panels, magenta) fractions of tubules positive for both tubular injury marker (Krt8) and loop of Henle (Umod) or distal tubule/collecting duct (DBA) marker.

**Figure 7:**
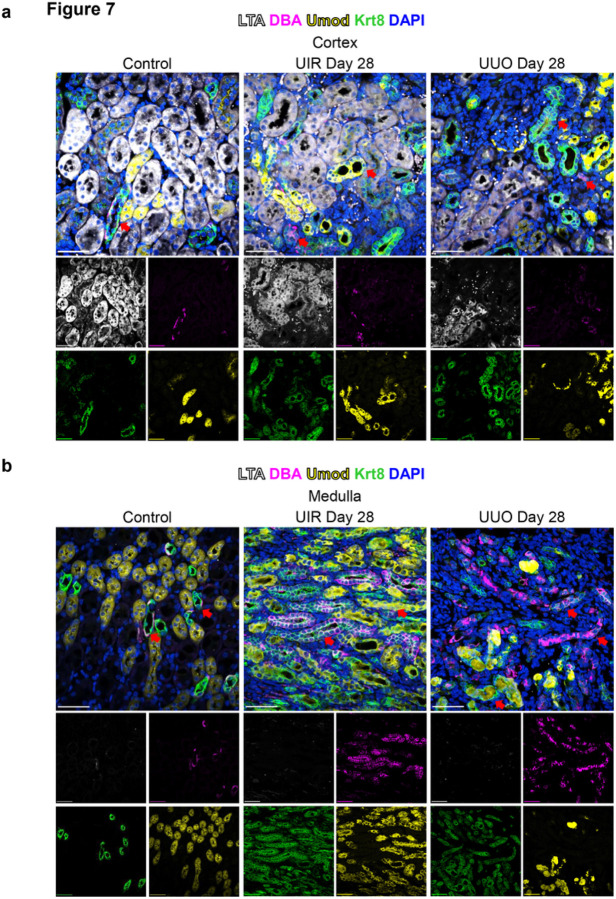
Distal segments of the nephron tubule exhibit persistent Krt8 elevation at advanced kidney fibrotic remodeling stages. (a) Representative images of combined Krt8 IF (green), Umod (yellow), DAB (magenta), LTL (white) and DAPI (blue) in the cortical areas of control, UIR and UUO kidneys. Original magnification, ×60, 0.14 μm/px zoom. Areas of Krt8 co-localization with Umod or DAB are shown with red arrows. (b) Representative images of combined Krt8 IF (green), Umod (yellow), DAB (magenta), LTL (white) and DAPI (blue) in the medullary areas of control, UIR and UUO kidneys. Original magnification, ×60, 0.14 μm/px zoom. Areas of Krt8 co-localization with Umod or DAB are shown with red arrows.

We noted that while both models of advanced kidney injury caused decrease of the PT populations, the remaining PTs restored normal mature gene expression signatures by Day 28 (Supplementary figures S6–9). Thus, we sought to elucidate the transcriptional states of other tubular segments. scRNA-seq demonstrated that while the clinically recognized tubular injury marker *Lcn2*^54^ was elevated in the loop of Henle and distal tubule of fibrotic kidneys, neither model exhibited elevation of *Havcr1*, an established marker of PT injury,^55^ in any tubular clusters (Fig. 6a). Moreover, we revealed that other established markers of failed tubular repair, such as *Spp1*^56^ and *Krt7/8/18/*19^57^ were predominantly enriched in the distal segments of nephron tubule, sparing the surviving PTs (Fig. 6a). This spatial tubular injury pattern was validated with

immunofluorescence (IF), which demonstrated increased Krt8 levels in Uromodulin (Umod)-positive loop of Henle and Dolichos biflorus agglutinin (DBA)-positive distal tubule/collecting duct of both UIR and UUO kidneys (Fig. 6b). While Krt8 elevation marked both cortical and medullary segments of loop of Henle, distal tubules and collecting ducts, the remaining Lotus tetragonolobus lectin (LTL)-positive PTs were spared (Fig. 7a and b). Thus, our data shows that distal segments of the nephron tubule endure persistent and unresolved injury throughout the course of advanced kidney fibrotic remodeling.

### Kidney fibrosis causes renal developmental program reactivation in the stromal and distal nephron tubular populations.

**Figure 8:**
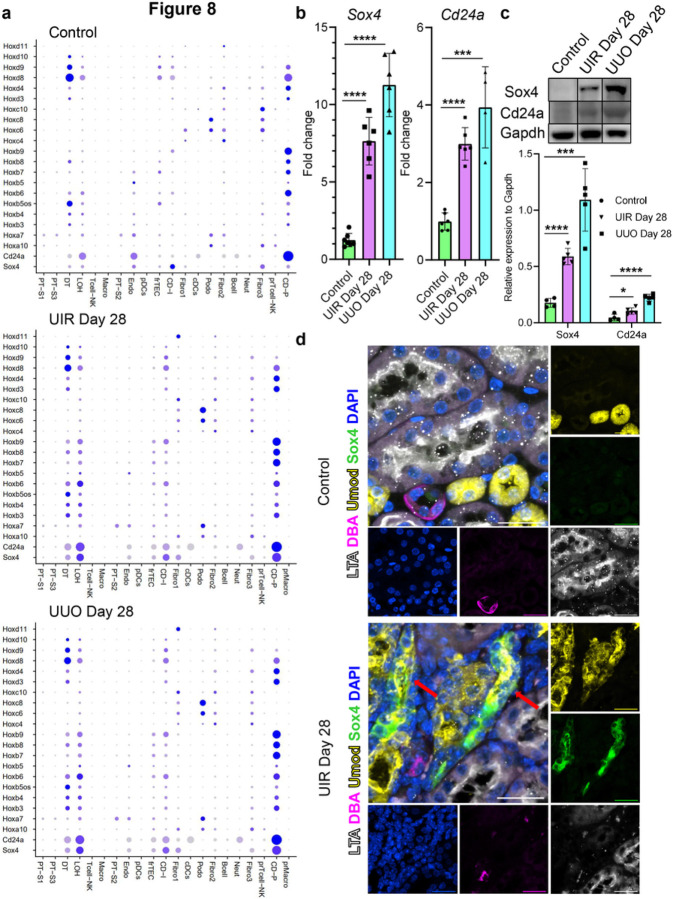
Advanced fibrotic injuries cause renal developmental program reactivation in the distal nephron tubular segments of adult kidney. (a) Dot plot of cell type-specific expression of renal developmental genes for manually annotated clusters in the control, UIR and UUO kidney. Dot size denotes percentage of cells expressing the marker. Color intensity represents average gene expression values. (b) *Sox4* and *Cd24a* qPCR in control and fibrotic kidneys, n=4–7 per group. (c) Representative bands and quantifications of Sox4 and Cd24a Western blots, n=4–5 per group. Representative bands are cropped out of the original gels and are separated by the black border, the unprocessed original blots/gels are presented in Supplementary figure S26. (d) Representative images of combined Sox4 IF (green), Umod (yellow), DAB (magenta), LTL (white) and DAPI (blue), control and UIR kidneys. Original magnification, ×60, 0.09 μm/px zoom. Areas of Sox4 colocalization with Umod are shown with red arrows.

Among the molecular changes underlying pathologic long-term kidney remodeling, we noted robust reactivation of genes normally expressed during nephrogenesis, including *Hox* genes (Fig. 8a).^58^*Hoxb3/4/6/7/8/9, Hoxd3/4* were upregulated in distal segments of the UIR and UUO nephron tubule, while *Hoxd11* was elevated in Fibro 1 clusters of fibrotic kidneys (Fig. 8a). Of note, we found that some *Hox* gene expression is present even in the adult normal kidney, mostly in the principal cells. Our data also showed elevation of renal developmental genes *Cd24a* and *Sox4* in both UIR and UUO. We recently reported that AKI elicited increased *Sox4* and Cd24a expression, which returned to baseline as the injury resolved.^29^ However, scRNA-seq revealed persistent upregulation of these nephrogenic genes in both models of AKI-to-CKD transition, which was validated in additional UIR and UUO cohorts at the RNA and protein levels (Fig. 8b and c, Supplementary figure S26). Of note, both renal developmental genes were elevated in the distal nephron tubular segments, frTECs and fibroblasts, sparing the PTs. IF corroborated scRNA-seq findings, identifying that fibrosis caused Sox4 elevation in the loop of Henle, sparing the remaining LTL-positive PTs (Fig. 8d). Collectively, we revealed that AKI-to-CKD transition causes persistent nephrogenic signaling reactivation in multiple populations, including distal segments of the nephron tubule.

## Discussion

This study presents a single cell model-specific transcriptional profiling of fibrotic CKD. With combination of scRNA-seq and thorough validation, we reveal key cellular and molecular mechanisms of long-term kidney remodeling and a novel putative kidney fibroblast marker.

We previously created a thorough transcriptional profiling of AKI recovery.^29^ The current study focuses on maladaptive long-term kidney injury response in two clinically relevant murine models of AKI-to-CKD transition. As our previous report suggests, first signs of kidney fibrosis and maladaptive responses develop on Day 14 after the injury induction. Thus, Day 28 was chosen for this study to ensure the complete onset of advanced fibrosis and kidney remodeling. UIR and UUO Day 28 repeatedly displayed key CKD features, such as kidney parenchymal reduction and functional blood flow decline. scRNA-seq performed on multiple replicates with two independent platforms showed dramatic PT loss, inflammatory infiltration, and stromal expansion in both models, which was validated in separate control, UIR and UUO cohorts.

Both models elicited three novel fibroblast clusters, consistent with recent reports revealing kidney stroma heterogeneity.^59^ Particularly, the recent study by Kuppe et al.^23^ used sorting to isolate PT and non-PT fractions from hypertensive CKD patients and dissect the heterogeneity of renal interstitium. Consistent with their human findings, we identified that murine CKD results in higher ECM related gene expression compared to the control. We found that while both UIR and UUO induce crucial CKD pathological landmarks, UUO causes more robust renal blood flow decline, tubular injury, inflammation and epithelial parenchymal remodeling. Thus, we established two independent models allowing to simultaneously examine the molecular and cellular changes in the fibrotic kidney with respect to the injury cause and severity. Among the non-PT fraction, Kuppe et al. identified the mesenchymal populations, including *Postn*-myofibroblasts, *Dcn*-positive fibroblasts and *Cox4i2*-positive pericytes, all exhibiting high ECM related gene expression score. Consistent with that, we observed ECM and collagen fiber organization related gene enrichment in the Fibro 1 population, thus annotating it as the most responsible for fibrotic remodeling. However, we observed that while all three of scRNA-seq identified fibroblast clusters expressed *Pdgfrβ*, Fibro 1 was the only fibroblast fraction labelled by *Dcn* and *Col1a1*. Thus, our data suggests that those ECM related genes might not be used to comprehensively label kidney fibroblasts. On the contrary, Fibro 2 was the only population which elevated classic myofibroblast marker *Acta2*, thus we labelled them as contractile. We noted that Fibro 3 elevated pericyte markers^60^*Pdgfrβ* and *Dsm* relative to other fibroblasts. Moreover, control, UIR and UUO Fibro 3 exhibited increased *Pdgfrα* recently implicated in vascular fibrosis.^61^ While Fibro 1 and 2 clusters were expanded in UIR and UUO compared to the control, Fibro 3 represented major stromal fraction in the normal kidney. Overall, our findings contribute to understanding the heterogeneity of kidney stroma and highlight the need for a specific marker which would allow for thorough labeling and targeting of all activated kidney fibroblasts with no off-target expression.

We also show that both models cause significant PT dropout compared to the control, with UUO causing near-total PT loss and more aggressive fibrosis than UIR, which is consistent with a recent report^59^ which also identified diverse PT injury states and repair outcomes: UUO Day 14 elicited large aberrantly repaired PT fraction and persistent healthy PTs decline, while UIR Day 28 exhibited near-total repair. Instead, we found that both UIR and UUO at Day 28 exhibit persistent PT decline. This divergence in the UIR response might reflect the differential effects of ischemia duration on the PT injury.

We found that loop of Henle, distal tubule and collecting duct exhibit the highest levels of epithelial developmental and injury related genes which persisted at Day 28, while the surviving PTs displayed restored mature gene expression. Moreover, frTECs exhibited transcriptional similarity with embryonic and adult distal segments of the nephron tubule, showing little transcriptional overlap with PTs. Of note, we found that *Sox4*, recently reported in the human AKI urine using scRNA-seq,^62^ was strongly elevated in UIR and UUO loop of Henle, distal tubule and principal cells. Our findings highlight the previously unrecognized salutary response of the distal nephron in kidney fibrosis, which may be targeted for diagnostic and therapeutic interventions.

## Methods

### **Animals**.

The Institutional Care and Use Committee (IACUC) of Cincinnati Children’s Hospital Medical Center approved all animal procedures in the study. All the experiments and methods, including animal husbandry and monitoring, were performed in accordance with relevant IACUC guidelines and regulations. Data reporting in the manuscript follows the recommendations in the ARRIVE guidelines.^63^ Unilateral ischemia/reperfusion (UIR) was induced via atraumatic left renal pedicle clamping for 30 minutes at 37°C and unilateral ureter obstruction (UUO) was induced via irreversible left ureter ligation in 10 weeks old male C57Bl/6 mice (n = 3 for 10x Chromium scRNA-seq, 1 for Drop-seq per model). Mice were anesthetized by 3% Isoflurane anesthesia gas before the procedures and received 1.5–2% Isoflurane anesthesia gas during operating. Buprenorphine sustained release (SR) was administered after operating 0.5–1mg/kg subcutaneously. The kidneys were harvested at Day 28 post-injury. For euthanasia, mice were exposed to overdose inhalant anesthetic (Isoflurane), followed by exsanguination and organ harvest. Naive mice of the same age, strain and sex (n = 5) were used as controls.

### Sample preparation and scRNA-seq analysis.

Single cell suspensions were prepared with *Bacillus licheniformis* cold active protease^64^ and sequenced using an Illumina Novaseq 6000 following the 10X Genomics protocol for library construction using the Single Cell 3’v3 chemistry. The fastq files were processed using 10x Genomics Cell Ranger v6.1.2 and ambient RNA was mediated by using the decontX function within the celda package.^65^ Resulting datasets were further cleaned using doubletFinder package^66^ with 7.5% doublet occurrence per data set. For details, see the Supplemental Methods.

### Equipment and settings.

Macroscopic kidney images were obtained on Zeiss Axiovert 25 wide-field microscope. Magnetic resonance imaging (MRI) was performed using a horizontal 7T Biospec MRI system (Bruker, Billerica, MA); axial images were acquired using a fast spin echo sequence with a repetition time of 2500 ms, echo time of 40.2 ms, echo train length of 16, 4 averages, 32 mm × 32 mm field of view, and an acquisition matrix of 200 × 200. IF images were produced on Nikon Ti-E A1R HD confocal with the resonant scanner, processed with NIS-Elements AR 5.2.00 artificial intelligence denoise algorithm and analyzed with Bitplane Imaris 9.9.0.^29^ All images within an experimental group were obtained and displayed with the same optical configurations. Western blot imaging was performed using a ChemiDoc imaging system and Bio-Rad’s Image Lab Touch Software, quantitative analysis was done in ImageJ. For details on the used antibodies, including Sox4^67,68^ and Cd24a^29,69^, see the Supplemental Methods.

### Statistical analysis.

scRNA-seq was reproduced in three independent runs using DropSeq and 10x Chromium platforms; validation was performed on leftover scRNA-seq suspensions and separate UIR, UUO and control mice (n = 4–6 per group). *P* values were generated using Student’s t-test with *p < 0.05 representing the statistically significant difference. Data are presented as individual values, mean ± SD.

## Data Availability

The datasets generated and/or analyzed during the current study are available at the Gene Expression Omnibus under accession number GSE198621.
